# The Public Health

**Published:** 1918-08-10

**Authors:** 


### THE PUBLIC HEALTH.

### Some Topical Notes.

### Ministry of Public Health.

The urgent question of a Ministry of Public Health is receiving increased attention both in the medical and public press. The pulse of popular feeling clearly indicates that the establishment of such a Ministry is considerably overdue, while the national requirements demand that the problems relating to public health and the prevention and treatment of disease should be unified and co-ordinated under one responsible authority. The ravages of the war and the falling birth-rate have increased the necessity of conserving and preserving the health and lives of adults and children, and no minor difficulties should be allowed to stand in the way of a measure which is not only of national importance, but which is clearly called for by the expression of public opinion.

### Cerebro-Spinal Fever.

Cases of cerebro-spinal fever continue to crop up in various districts. The careful swabbing of all contacts is an essential preventive measure, but the percentage of such contacts which prove to be carriers of the meningococcus is usually very low; occasionally, however, positive results are obtained in a considerable percentage of cases. The carrying out of open-air measures and continued naso-pharyngeal antisepsis are essential in the treatment of carriers. The prompt use of anti-meningococcic serum is necessary if the high case mortality from this disease is to be substantially reduced. County and county borough authorities are now empowered to arrange for the provision of serum, and for the apparatus necessary for its use. Such measures, combined with the arrangements which exist to facilitate the diagnosis of the disease by expert examination of the cerebro-spinal fluid, should lead to more promising results in the prevention and treatment of cerebro-spinal fever.

### Epidemic Diarrhœa.

If the weather continues warm and dry during the next few weeks special measures will be necessary with regard to the prevention and treatment of epidemic diarrhœa. The importance of such measures is indicated by the fact that the Local Government Board has issued a special memorandum on the subject. The memorandum points out that the conditions arising from the war are calculated to increase the risk to young children from this very fatal disease. Mothers are less occupied with domestic duties and the supervision of home and food; scavenging and the removal and destruction of refuse are less frequently and less efficiently carried out, consequently the fly plague will in no way be diminished unless the barometer is low during the next few weeks. As the saving of child-life at the present time comes next in importance to winning the war, special efforts are therefore called for on the part of health visitors and sanitary inspectors with regard to preventive measures, and on the part of hospital authorities to make some provision for the treatment of such cases of epidemic diarrhœa as cannot be treated at home with any prospect of recovery.

